# IT’S A FAMILY AFFAIR: ADDRESSING THE NEEDS OF BLACK DEMENTIA CAREGIVERS THROUGH COMMUNITY-BASED RESEARCH

**DOI:** 10.1093/geroni/igad104.0754

**Published:** 2023-12-21

**Authors:** Dianne Oladejo, Joyce Chapman, Tonya Roberson, Rachel O’Conor

**Affiliations:** Northwestern University, Chicago, Illinois, United States; Far South Chicago Coalition, Chicago, Illinois, United States; Governors State University, Chicago, Illinois, United States; Northwestern University, Chicago, Illinois, United States

## Abstract

Racial disparities exist in dementia diagnosis, co-morbid health conditions, and socioeconomic characteristics between older Black and White adults, compounding caregiving responsibilities for Black families; yet few dementia caregiving programs offer culturally competent, family-centered dementia caregiving programs for Black individuals. In a collaboration between two community-based organizations (CBO) and an academic medical center, a culturally tailored, family-centered, dementia caregiving program for Black individuals on Chicago’s Far South Side was proposed. Local CBOs convened community members to co-design a key informant interview guide and conducted 30 interviews among Black dementia caregivers. The caregivers interviewed were primarily caring for parents or grandparents and shared caregiving responsibilities with other family members. Caregivers recommended the program focus on five top concerns related to their caregiving experience: 1) feelings of stress and burden while being committed to their family member with dementia, 2) education on the progression of dementia and discussion of cultural beliefs, 3) personal experiences with racism and intersectional identities, 4) kinship responsibilities and managing dementia-specific changes that occur with loved ones, and 5) a need for respite and local medical and community-based resources. Anticipated barriers to participation in this program included time, an accessible location to members of the far south, and care for family members during participation. Though a community-directed approach, we elevated Black dementia caregiver experiences to better understand the influence that race and culture has on Black caregivers, which will provide the foundation for a Black family-centered dementia caregiving program to build capacity in routine caregiving and reduce caregiver stress.

